# Supplementary material for: Captive chimpanzee foraging in a social setting: a test of problem solving, flexibility, and spatial discounting
Source: PeerJ. 2015 Mar 17;3:e833. doi: 10.7717/peerj.833 (PMC4369338; doi:10.7717/peerj.833)
Supplement: Supplemental Information 1 — A comparison of the average number of exchanges made by the chimpanzees at the CLOSE and FAR locations, for each of the three phases, across the different categories of zoo visitor numbers present for each test session. As exchange location B (Fig. 1) was the closest to the visitor viewing area (5.7 m away at the closest point), it might be anticipated the chimpanzees would avoid exchanging tokens at this location when higher numbers of visitors were observing the test sessions. However, there was no influence of the number of visitors present at the exhibit on the locations where the chimpanzees exchanged tokens. (We recorded the number of visitors in front of the exhibit at the start of each test session using a categorical scale where “0”, only staff present; “10”, 1–10 visitors around the exhibit; “20”, 11–20 visitors around the exhibit; “30”, 31–40 visitors around the exhibit; “40”, 41–50 visitors around the exhibit; and “50”, 41–50 visitors around the exhibit. In phase 1, the chimpanzees experienced conditions 10, 20, and 30. In phase 2, they experienced conditions 0, 10, 20, 30, and 40. In phase 3, they experienced conditions 0, 10, and 30.) We also considered that the chimpanzees’ exchanges for grapes might have been influenced by each chimpanzee’s individual food preferences. To test this, we compared the proportion of rewards that each chimpanzee ate in the food preference tests that were grapes (i.e. the strength of each chimpanzee’s preference for grapes) and their exchange behavior. There was no correlation between the chimpanzee’s pre-test preference for grapes and the number of tokens that they exchanged for grapes (Spearmans’s rho: rs = 0.313, N = 6, P = 0.545) or the number of tokens they exchanged for carrots (rs = 0.441, N = 6, P = 0.381) across the three phases combined. This is likely because all the chimpanzees showed comparably strong preferences for grapes and so this did not predict the variation in their participation in this study or where [file peerj-03-833-s001.docx]

| **Phase** | **CLOSE** | **FAR** |
| --- | --- | --- |
| **1** | X^2^(2) = 1.37, P = 0.504 | X^2^(2) = 0.18, P = 0.913 |
| **2** | X^2^(5) = 2.60, P = 0.761 | X^2^(5) = 2.20, P = 0.821 |
| **3** | X^2^(3) = 6.96, P = 0.073 | X^2^(3) = 3.00, P = 0.392 |
