# Supplementary figures and images for: Captive chimpanzee foraging in a social setting: a test of problem solving, flexibility, and spatial discounting
Source: PeerJ. 2015 Mar 17;3:e833. doi: 10.7717/peerj.833 (PMC4369338; doi:10.7717/peerj.833)

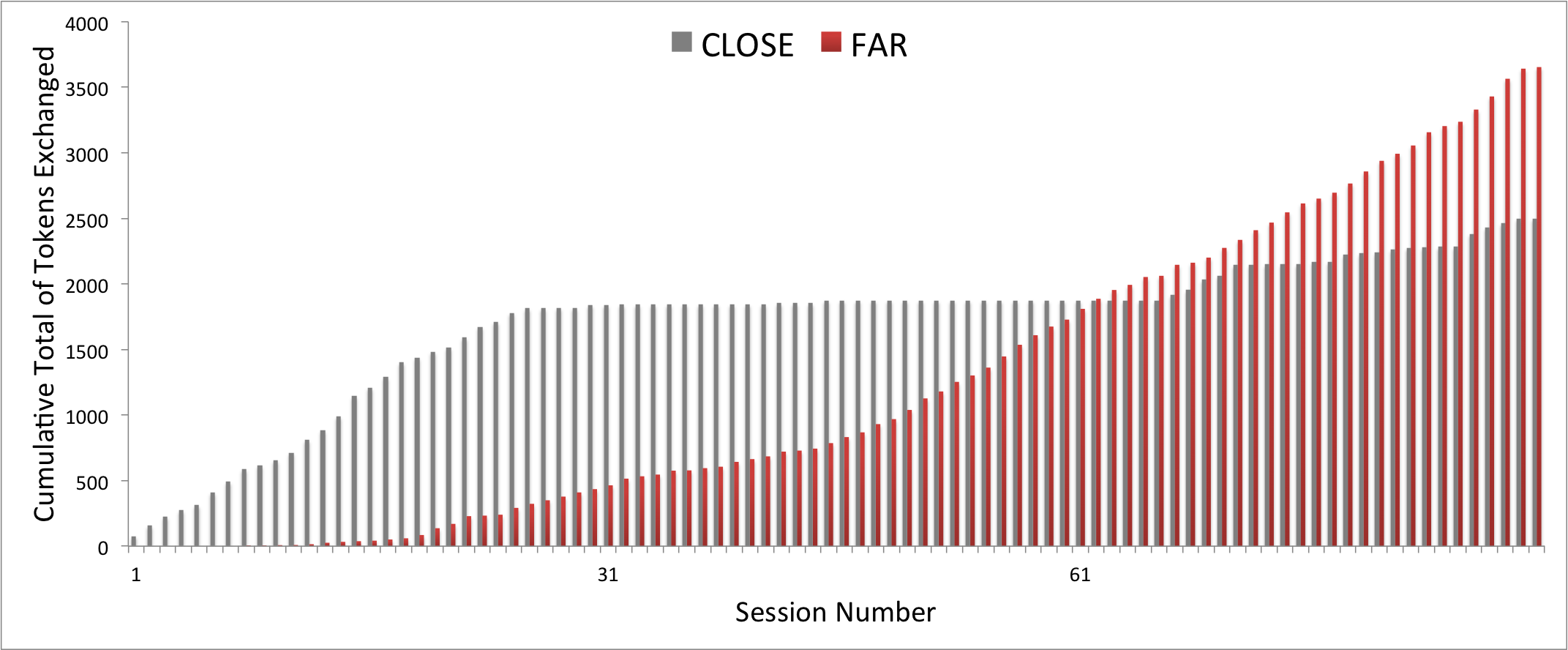

Supplement: Supplemental Information 2 — The chimpanzees first exchanged a token at the CLOSE location during the first session and made their first exchange at the FAR location during the eight session. Note the plateau of exchanges made during phase 2 (sessions 31–60) when the chimpanzees had to travel to obtain either reward. [file peerj-03-833-s002.png]
